# The use of external fixation for the correction of recurrent clubfoot: a systematic review and meta-analysis

**DOI:** 10.1007/s00402-025-05776-1

**Published:** 2025-02-11

**Authors:** Marco Simone Vaccalluzzo, Gianluca Testa, Andrea Sodano, Marco Sapienza, Federico Canavese, Domenico Costantino Aloj, Alessia Caldaci, Vito Pavone

**Affiliations:** 1 University of Catania, Catania, Italy; 2 Department of Orthopedic and Traumatology, IRCCS Istituto Giannina Gaslini, Via Gerolamo Gaslini, 5, Genoa, Italy; 3 Dipartimento di scienze chirurgiche e diagnostiche integrate, University of Genova, Viale Benedetto XV N°6, Genova, Italy; 4 Ospedale Sant’Andrea, Vercelli, Italy

**Keywords:** Clubfoot, External fixation, Ilizarov technique, Recurrence, Pin tract infections, Meta-analysis

## Abstract

**Background:**

Recurrent clubfoot (CF) remains a challenging orthopedic condition, often requiring surgical intervention due to deformity rigidity and scarring from previous treatments. External fixation, particularly the Ilizarov technique, has emerged as a promising approach to correct recurrent and complex CF deformities. However, there is considerable variability in reported results regarding success and recurrence rates.

**Objective:**

This systematic review and meta-analysis aimed to evaluate the efficacy and safety of external fixation with a focus on success rates, recurrence rates, and complication profiles in patients treated for recurrent CF.

**Methods:**

A systematic search of five databases (PubMed, Embase, Web of Science, ScienceDirect, Cochrane Library) was performed according to PRISMA guidelines. Studies evaluating external fixation for recurrent clubfoot and reporting quantitative data on success and recurrence rates were included. A total of 438 records were screened and 21 studies met the inclusion criteria. Data extraction was performed on demographic characteristics, treatment details, and outcomes. Meta-analysis was performed using a random effects model to pool success and recurrence rates.

**Results:**

The review analyzed 21 studies involving 489 treated feet in 406 patients. The pooled overall success rate was 81.4% (95% CI: 74.5-88.4%), while the pooled recurrence rate was 17.7% (95% CI: 11.3-24.1%). The studies showed minimal heterogeneity (I² = 0%) in both success and recurrence rates. Complications were common, with pin tract infection being the most common (29.3%), followed by toe contractures and digital ischemia.

**Conclusions:**

External fixation is an effective approach for recurrent CF, with satisfactory success rates. However, the risk of complications underscores the need for vigilant postoperative care. The results support the use of external fixation for complex CF recurrences, but further studies are needed.

## Introduction

Congenital clubfoot (CF) or congenital talipes equinovarus is a complex deformity with various etiologies [1–3] and is commonly treated with serial casting using the Ponseti method. Although the Ponseti method is widely considered the gold standard for the treatment of CF due to its high success rate and minimal invasiveness ​ [3–5], recurrence is still a major concern, especially in patients who present late, have more severe deformities, or suffer from genetic or syndromic conditions [4, 6]​.

Clubfoot can present with different etiologies. Idiopathic clubfoot is the most common and occurs in isolation, without systemic associations. Syndromic clubfoot, on the other hand, is associated with conditions such as arthrogryposis or myelomeningocele, which contribute to increased rigidity and complexity. Post-traumatic clubfoot results from injuries or developmental disruptions and represents a less common but equally challenging variant [1–4, 6].

The treatment of recurrent clubfoot CF presents significant challenges due to the rigidity of the deformity and the presence of scar tissue from previous procedures. While additional serial casting can improve the deformity, stretch the soft tissues and be effective in some cases, more severe recurrences often require surgical intervention. Historically, surgical procedures for recurrent CF have included soft tissue releases, tendon transfers, and bony osteotomies​ [4, 7]. Despite the effectiveness of these methods [4, 8], complications and high recurrence rates have led to the development and increased use of circular external fixation, which allows gradual, simultaneous, multiplanar correction of the deformity while allowing precise adjustments over time​ [7, 9].

The gradual correction provided by external fixation minimizes the risks associated with more acute corrective procedures, such as neurovascular injury, overcorrection, and residual deformity, and allows weight bearing during treatment, which is particularly beneficial in older children and adults​ [4–6].

However, the use of external fixation is not without complications, including pin tract infections and toe contractures. Pin tract infections can lead to significant morbidity, including loosening of the pins or, in severe cases, removal of the frame, if not treated promptly [5], while toe contractures can occur with prolonged fixation​ [4, 5].

Given the variability in reported outcomes, ranging from success rates of 70–90% and recurrence rates of 15–50% [9–12], there is a substantial need for a systematic review and meta-analysis to provide a comprehensive evaluation of the efficacy and safety of external fixation for treating recurrent CF.

The objective of this systematic review and meta-analysis is to pool data from multiple studies to assess the overall success and recurrence rates associated with external fixation for the treatment of recurrent CF and to quantify the incidence of complications such as infection and contractures. In addition, we seek to evaluate the impact of adjunctive procedures, such as osteotomies and tendon transfers, on the long-term outcomes of patients treated with external fixation.

## Materials and Methods

### Search Strategy

A comprehensive literature search was conducted up to September 2024 using PubMed, Embase, Web of Science, ScienceDirect, and the Cochrane Library databases and included retrospective, prospective, and longitudinal cohort studies.

Three junior researchers (MSV, MS, GT) independently screened titles and abstracts and extracted data according to predefined inclusion and exclusion criteria; editorials and letters to the editor were also screened. To identify potential missing articles, the references of all selected articles were also checked. All articles considered relevant after the initial screening were retrieved and assessed for eligibility. All ineligible articles were excluded and duplicate articles were removed.

Disagreements between junior and senior authors (GT and FC) regarding the inclusion and exclusion criteria of a publication were resolved by consensus. Our analyses followed the Preferred Reporting Items for Systematic Review and Meta-analysis (PRISMA) guidelines for a systematic review of success rates.

### Search terms and delimiting

Search terms included “External Fixation” OR “Ilizarov” OR “External Fixators” OR “Orthopedic Fixation Devices” AND “Clubfoot” OR “Talipes” AND “Recurrence” OR “Relapse” OR “Failure” OR “Revision Surgery” including combinations of the index and free-text terms, as recommended in the Cochrane Handbook for Systematic Reviews of Interventions. The search was restricted to the English language and human participants.

### Selection criteria

Study inclusion criteria were as follows: (1) studies of patients with recurrent CF treated with external fixation techniques, specifically including the Ilizarov method and the other orthopedic external fixation devices; (2) studies reporting quantitative data on success rates, recurrence rates, or both; (3) studies with a follow-up of at least 6 months; (4) articles published in English; (5) full article text available; and (6) studies with a Newcastle-Ottawa Scale (NOS) quality score ≥ 6 points [13].

Exclusion criteria were as follows: (1) case reports, editorials, or review articles without original data; (2) studies not related to recurrent CF or external fixation; (3) studies with fewer than 10 patients; (4) articles on other foot conditions; (5) studies without sufficient quantitative data or with inadequate follow-up; (6) articles in languages other than English; and (7) studies with NOS quality score ≤ 6 points [13].

### Quality evaluation

The quality of the included studies was assessed using the NOS for cohort studies, which assesses three domains: selection of study groups, comparability of groups, and outcome assessment. Studies were scored on a scale from 0 to 9, with higher scores indicating better methodological quality [13]. In particular, studies with a score of 6 or higher were considered to be of high quality.

Two researchers independently (MSV, MS) graded the studies according to the NOS criteria checklist. In the case of conflicting scores for an article, the two reviewers discussed and proposed a consensus score.

### Literature screening and data extraction

The extracted data included: (1) characteristics of the included literature, including first author, year of publication, nationality, number of patients, time of case collection, and type of study; (2) clinical characteristics of patients, including age at diagnosis, sex, type of treatment, type of external fixator used (Ilizarov or Taylor Spatial Frame [TSF]), duration of treatment, need for additional surgical procedures, treatment success rate, incidence of complications (e.g., pin tract infection, toe contracture), and final outcome (success rate, defined as the percentage of feet achieving clinical and/or radiographic correction; recurrence rate, defined as the percentage of feet experiencing recurrence of deformity).

All included studies utilized the Ilizarov circular external fixator, with the exception of one study (Eidelman et al.), which employed the Taylor Spatial Frame (TSF), a circular external fixator designed for multiplanar corrections.

**Fig. 1:**
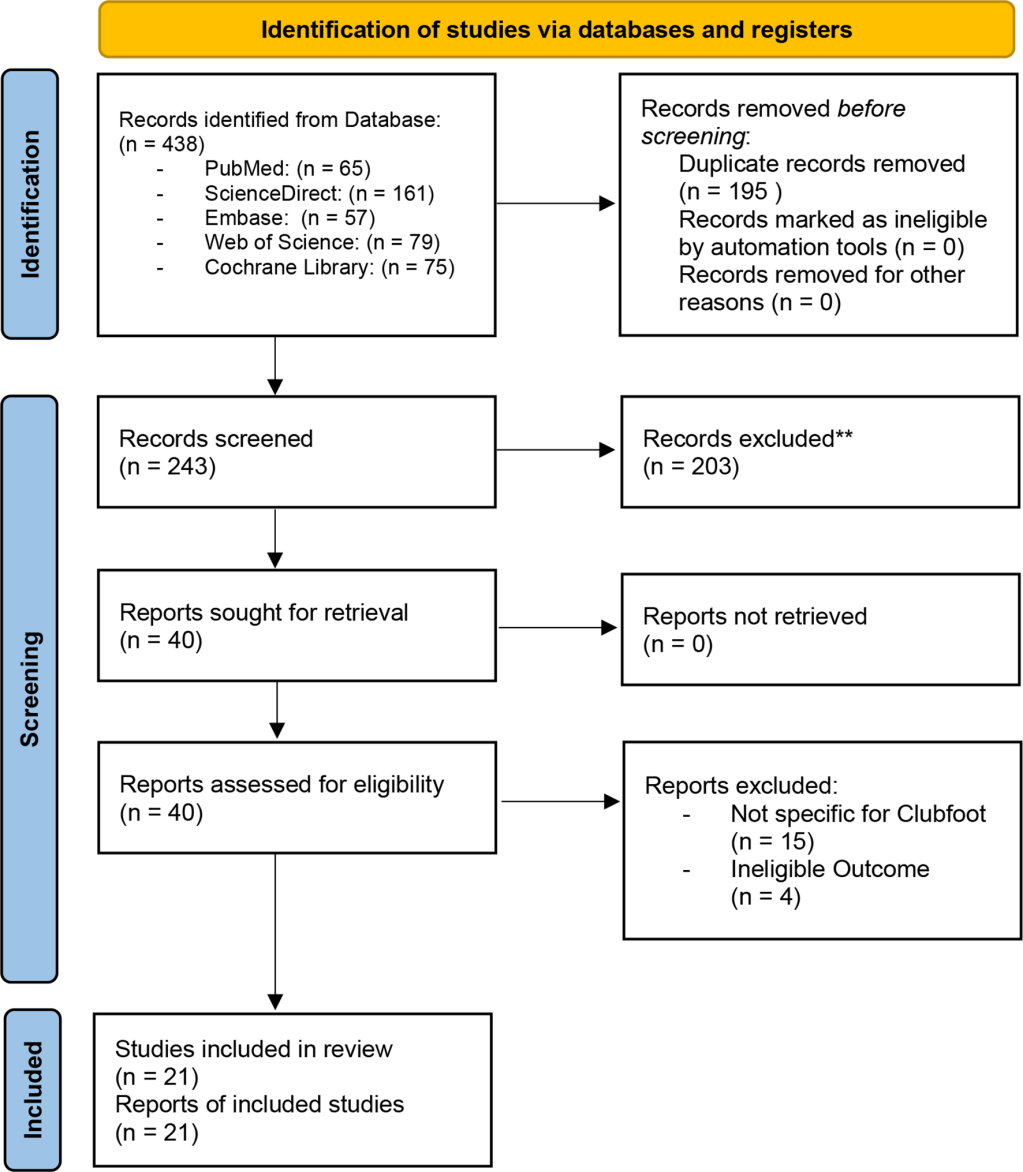
PRISMA 2020 flow diagram outlining the identification, screening, and inclusion process for the studies included in this systematic review

Figure 1 shows the overall search and study selection flowchart; of the 438 studies screened for eligibility, 195 duplicates were excluded. Of the 243 remaining papers, 203 were excluded based on title and abstract for irrelevant focus (e.g. not related to clubfoot or recurrence), not including external fixation or the Ilizarov technique, and outcome measures that did not address recurrence or success rates. Finally, 40 studies were included in the review (Fig. 1): 19 studies were further excluded because they were not specific to CF (*n* = 15) and because of ineligible outcome measures (*n* = 4). Finally, 21 studies met all inclusion criteria and were included in the final analysis.

The extracted data are summarized in Table 1, which is a comprehensive overview of the characteristics and outcomes of the 21 studies in the study (Table 1).

**Table 1:** Summary of Key findings from selected studies on the use of external fixation in Clubfoot Recurrence Treatment

Author	Number of Patients	Number of Feet Treated	Average Age (years)	Age Range (years)	Male (%)	Female (%)	Mean Follow-up (months)	Mean Treatment Duration (weeks)	Success Rate (% good/excellent)	Major Complications	Recurrence Rate (%)
Amit Sharma et al. [16]	23	30	8 ± 3.6	4–17	79.2	20.8	36	6 ± 1.7	76	Pin tract infection (93.3%)	0
Mohamed El-Sayed [17]	38	42	6	3–13	71	29	55.2	9.8	88	Pin tract infection (71.4%), plantar flexion (61.9%)	4.76% (2 patients)
Xiao Jian Wang et al. [11]	15	18	9 ± 3	4–13	73.3	26.7	22	23.8	72	Toe contracture (1), wire-hole infection (1), residual deformity (3), recurrence (2), spastic ischemia (1)	11.1% (2 patients)
Ricardo Cardenuto Ferreira et al. [18]	30	38	19	5–39	53.3	46.7	58	16	78.9	Pin tract infection, clawing toes (44.7%), digital ischemia	50% (19 patients)
Parmanand Gupta et al. [12]	15	16	7	4–14	86.7	13.3	27	12.2	87.5	Pin tract infection (62.5%), flexion contracture of toes	25% (4 patients)
Hani El-Mowafi et al. [19]	28	35	13.7 (osteotomy), 10.5 (soft tissue)	4–29	60.7	39.3	67.2	28	85.7	Pin track infection, mild residual varus and equinus in 5 feet	28.6% (10 patients)
Jason A. Freedman et al. [20]	17	21	5.7	2.7–9.8	64.7	35.3	79.7	27.62	14.3	Pin tract infections, recurrent deformity (52.4% required revision surgery)	52.4% (11 patients)
Xiao-Jian Wang et al. [21]	14	16	7.4 ± 3.6	3–13	64.3	35.7	20.1	25.5	81	Pin tract infection, toe contracture, residual deformity, spastic ischemia	12.5% (2 patients)
Konstantinos N. Malizos et al. [22]	12	13	7.8	3–17	75	25	63.7	11.4	77	Pin tract infection (12%), flat-top talus (3 feet)	14.3% (1 patient)
H. El Barbary et al. [23]	52	66	8.5	4–14	69.2	30.8	40	5.4	100	Pin-tract infection, wire cut-through, metatarsal length discrepancy	12% (8 feet)
Mohamed Ahmed Refai et al. [24]	18	19	8	4–15	66.7	33.3	54	5.5	84.2	Pin tract infection, toe contractures, flat-topped talus	15.8% (3 patients)
Hari Prem et al. [25]	14	19	5	2–8	64.3	35.7	82.8	16	74	Pin tract infection	5.3% (1 patient)
Ricardo Cardenuto Ferreira et al. [26]	29	35	14	4–31	51.7	48.3	56	10	77	Pin tract infection, toe flexion deformities (43%), spontaneous ankylosis (46%)	31% (11 patients)
Sujit Kumar Tripathy et al. [27]	12	15	7.3	4–10	75.0	25.0	30	4.2	66.7	Pin-tract infection, edema	0
Ashraf A. Khanfour [28]	21	25	10.9	9–13	81	19	43.2	21.65	84	No major complications	0
Henrik Wallander et al. [29]	7	10	6–15	6–15	100	0	40	10	60	Pin tract infection, anterior talus subluxation	No recidive, but reduced ankle joint motion in 5 feet
Mark Eidelman, Yaniv Keren, Alexander Katzman [30]	11	12	14.7	11–18	72.7	27.3	45	15.1	100	Pin-tract infection, premature consolidation	18% (2 patients)
Mehmet Kocaoglu et al. [31]	22	23	18.2	5–50	63.6	36.4	25	22.1	91.3	Pin-tract infection, toe contractures, metatarsal subluxation	4.3% (1 patient)
C. F. Bradish, S. Noor [9]	12	17	7.8	6–11	58.3	41.7	36	10.5	76.5	Pin-tract infection, toe flexion deformity	11.8% (2 patients)
J. Franke et al. [32]	12	13	11	7–15	66.7	33.3	60	9	100	Edema, Pin-tract infection, osteomyelitis	15.4% (2 patients)
Takanobu Nakase et al. [10]	4	6	7.4	4.5–10.5	50	50	61.2	8.9	83.3	Pin-tract infection, subtalar joint fusion	16.7% (1 patient)

### Statistical analysis

A meta-analysis was performed to pool the success and recurrence rates reported in the included studies. Success and recurrence rates were calculated using a random effects model (DerSimonian and Laird method [14]) to account for potential variability between studies due to differences in patient populations, treatment protocols, and follow-up periods. The random effects model was chosen to provide more conservative estimates in the presence of heterogeneity.

#### Pooled proportions

Success and recurrence rates were pooled by calculating weighted mean proportions. Each study’s contribution to the pooled estimate was weighted according to the inverse variance of its estimate, giving more weight to studies with larger sample sizes and more precise results.

#### Heterogeneity

The degree of heterogeneity between studies was assessed using the I² statistic, which quantifies the percentage of variation between studies that is due to heterogeneity rather than chance. An I² value of 0% indicates no observed heterogeneity, while values of 50% or more indicate substantial heterogeneity. In addition, Cochran’s Q test was used to test the null hypothesis that the observed variation was due solely to sampling error.

#### Confidence intervals

Pooled estimates of success and recurrence rates were presented with 95% confidence intervals (CIs), which provide a range within which the true population effect size is likely to fall with 95% confidence. Studies with more precise estimates (i.e., narrower CIs) contributed more to the pooled estimate.

#### Publication bias

A funnel plot was generated to assess the potential for publication bias. The funnel plot showed no significant asymmetry, indicating no evidence of publication bias. This was further confirmed by Egger’s regression test, which yielded a non-significant p-value (*p* > 0.05), suggesting that smaller studies did not disproportionately report more positive results.

All statistical analyses were performed using RevMan software. A significance level of *p* < 0.05 was used for all statistical tests. Forest plots were generated to visually represent the individual and pooled estimates of success and recurrence rates across studies.

## Results

In this comprehensive systematic review, 21 studies were analyzed to evaluate the use of external fixation for the correction of recurrent CF. A total of 489 feet (*n* = 406) were treated in these studies, with the majority of patients being male (*n* = 307/75.6%), reflecting the typical epidemiology of CF [8, 15]. The studies included both pediatric and adult populations, with the majority focused on pediatric cases (*n* = 365/89.9%). A smaller proportion of patients were adults (*n* = 41/10.1%).

### Demographic distribution

The included studies reported data on a total of 406 patients (*n* = 489 feet). The demographic characteristics of the study populations varied significantly in terms of age, gender distribution, and number of feet treated (Table 1).

- Gender distribution: In all studies, there was a male predominance in the patient population. A total of 307 male patients (75.6%) were treated compared to 99 female patients (24.3%), resulting in a male to female ratio of approximately 3.1:1. Some studies, such as Sharma et al., reported a particularly high proportion of male patients (79.2% male), while other studies, such as Nakase et al., reported a more balanced gender (50% male and 50% female).
- Age distribution: The age of patients ranged from 2 to 50 years. Most studies focused on pediatric populations, with the majority of patients being under 18 years of age. For example, El-Sayed et al. treated patients with a mean age of 10 years, while Cardenuto Ferreira et al. included both pediatric and adult patients and reported an age range of 6 to 45 years.
- Number of feet treated: The number of feet treated per study also varied widely, with some studies treating as few as 6 feet and others treating up to 66 feet. El Barbary et al. treated the highest number of feet (66 feet), while Franke et al. treated only 13 feet.

Table 1 provides a detailed breakdown of demographic characteristics for each study, including number of patients, number of feet treated, gender distribution, and age range (Table 1).

### Duration of follow-up

The duration of follow-up in the included studies ranged from 12 months to 82 months. Freedman et al. had the longest follow-up with a maximum of 82 months, while Refai et al. had the shortest follow-up of 12 months. The average follow-up across all studies was 48 months.

**Fig. 2:**
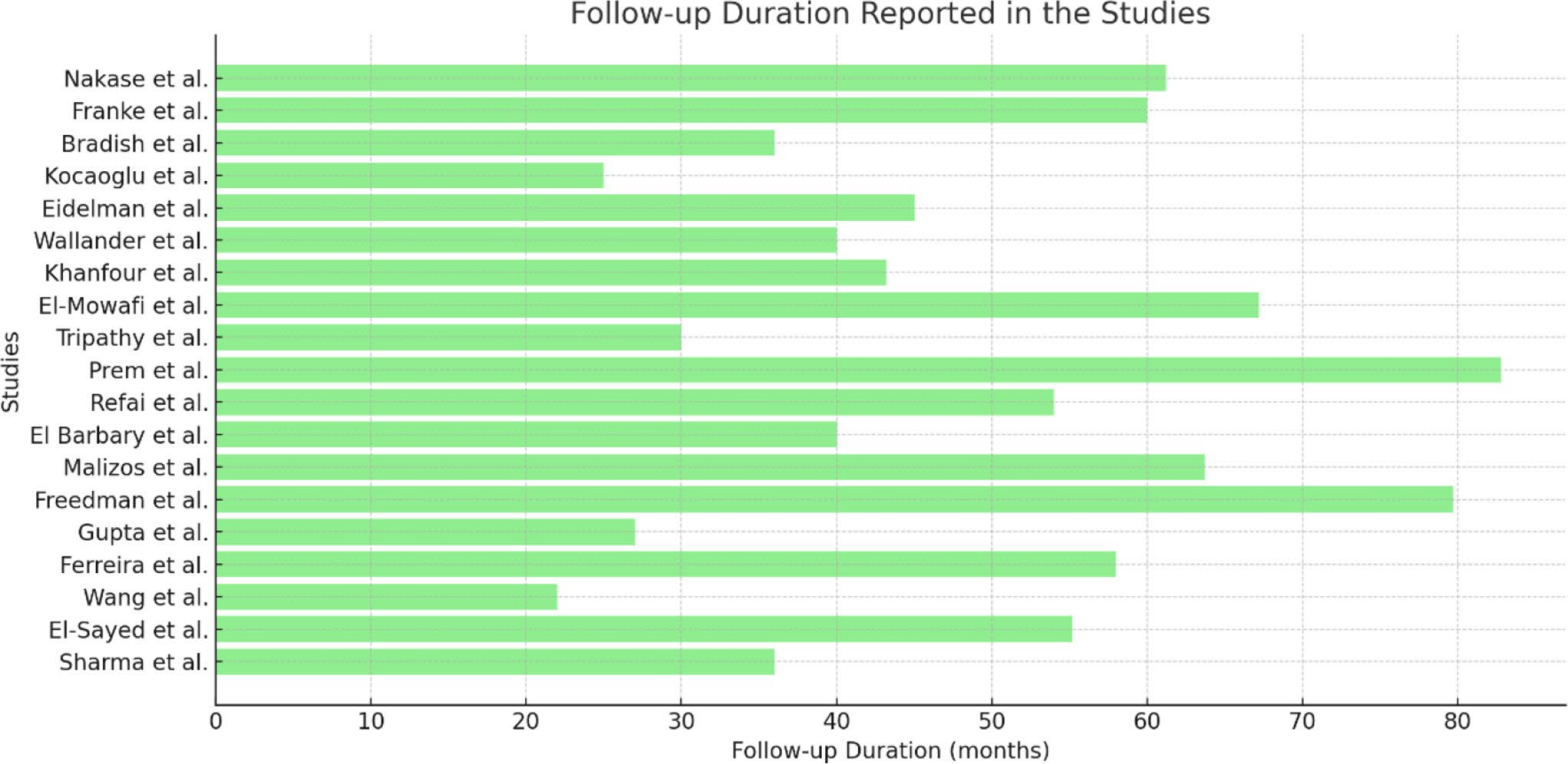
Follow-up Duration Reported in the Studies: Horizontal bar chart depicting the follow-up duration (in months) for each study. The variability in follow-up durations highlights the different observation periods across the studies, which may influence the reported rates of success and recurrence

The variability in follow-up time across studies is summarized in Fig. 2, which provides a visual comparison of the follow-up time for each study (Fig. 2).

### Success Rates

The success rates of the studies were quite variable, ranging from 14.3 to 100%. The study by El Barbary et al. reported the highest success rate of 100%, treating a total of 66 feet with an Ilizarov frame. On the other hand, Freedman et al. reported the lowest success rate of 14.3%, suggesting that more complex or resistant cases may benefit less from external fixation alone.

- Pooled success rate: The overall pooled success rate across all studies was 81.4% (95% CI: 74.5-88.4%), indicating that external fixation techniques generally provide satisfactory results in the majority of patients.
- Heterogeneity (I²): Statistical heterogeneity between studies was minimal, with an I² value of 0%. This suggests that the observed differences in success rates are largely due to random variation rather than differences in study populations or methods. The low heterogeneity implies that the pooled estimate is reliable and consistent across studies.

**Fig. 3:**
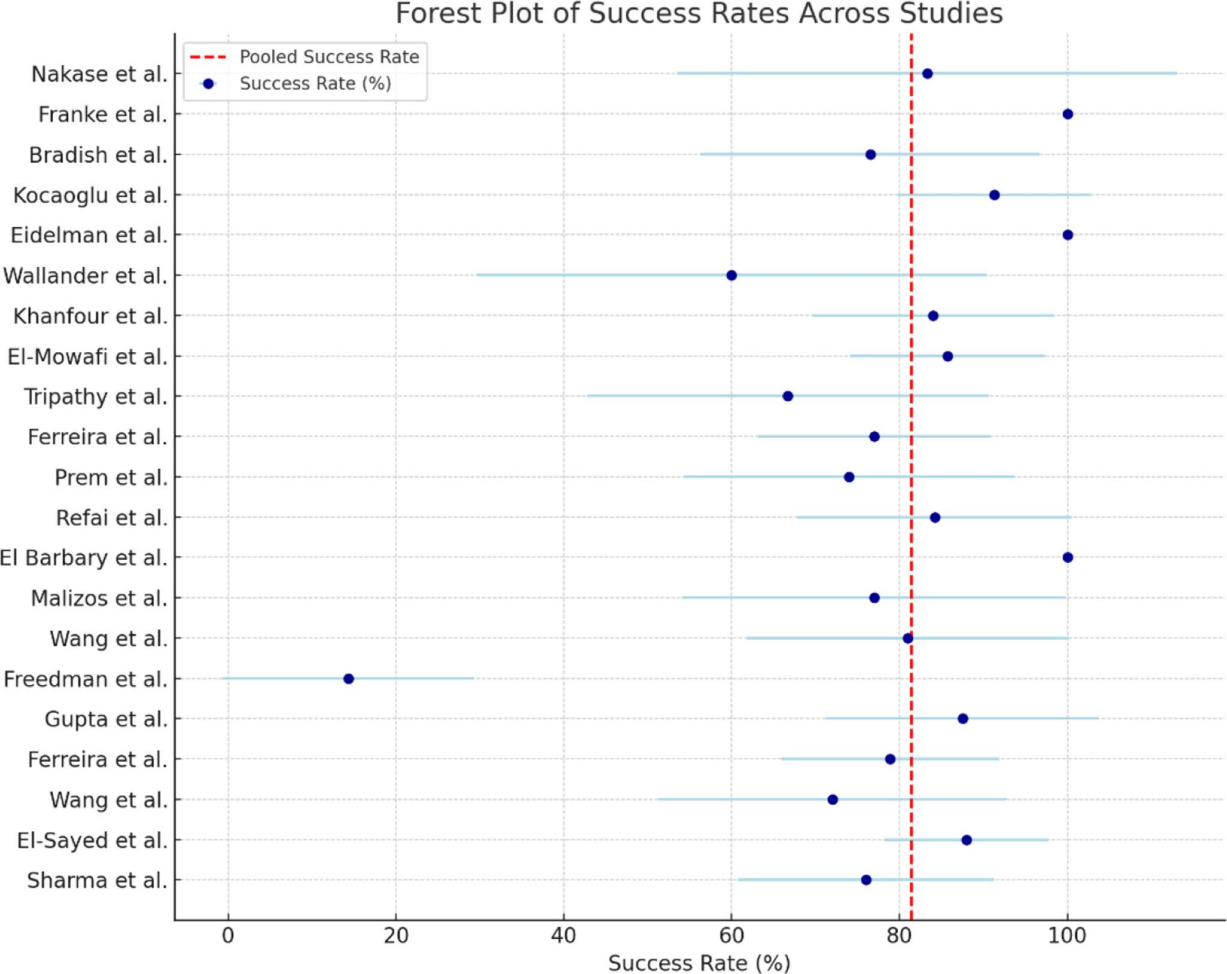
Forest Plot of Success Rates Across Studies: Forest plot illustrating the success rates with 95% confidence intervals for each study. The dashed line represents the pooled estimate of the success rate across all studies

The forest plot (Fig. 3) provides a visual representation of the success rates reported across studies, illustrating the range of results and confirming the overall high rate of successful corrections.

### Recurrence rates

Most studies reported recurrence rates ranging from 0 to 52.4%. Freedman et al. reported the highest recurrence rate of 52.4%, probably due to the complexity of the cases treated and the longer follow-up period. Conversely, Sharma et al. reported a 0% recurrence rate, suggesting that external fixation may provide durable correction in certain patient populations.

- Pooled recurrence rate: The pooled recurrence rate across all studies was 17.7% (95% CI: 11.3-24.1%). This suggests that, on average, approximately one in five patients may experience a recurrence following external fixation.
- Heterogeneity (I²): Similar to the success rate analysis, the heterogeneity for recurrence rates was low with an I² value of 0%. This indicates that the differences in recurrence rates between studies are primarily due to random variation rather than true differences in clinical practice or patient characteristics.

**Fig. 4:**
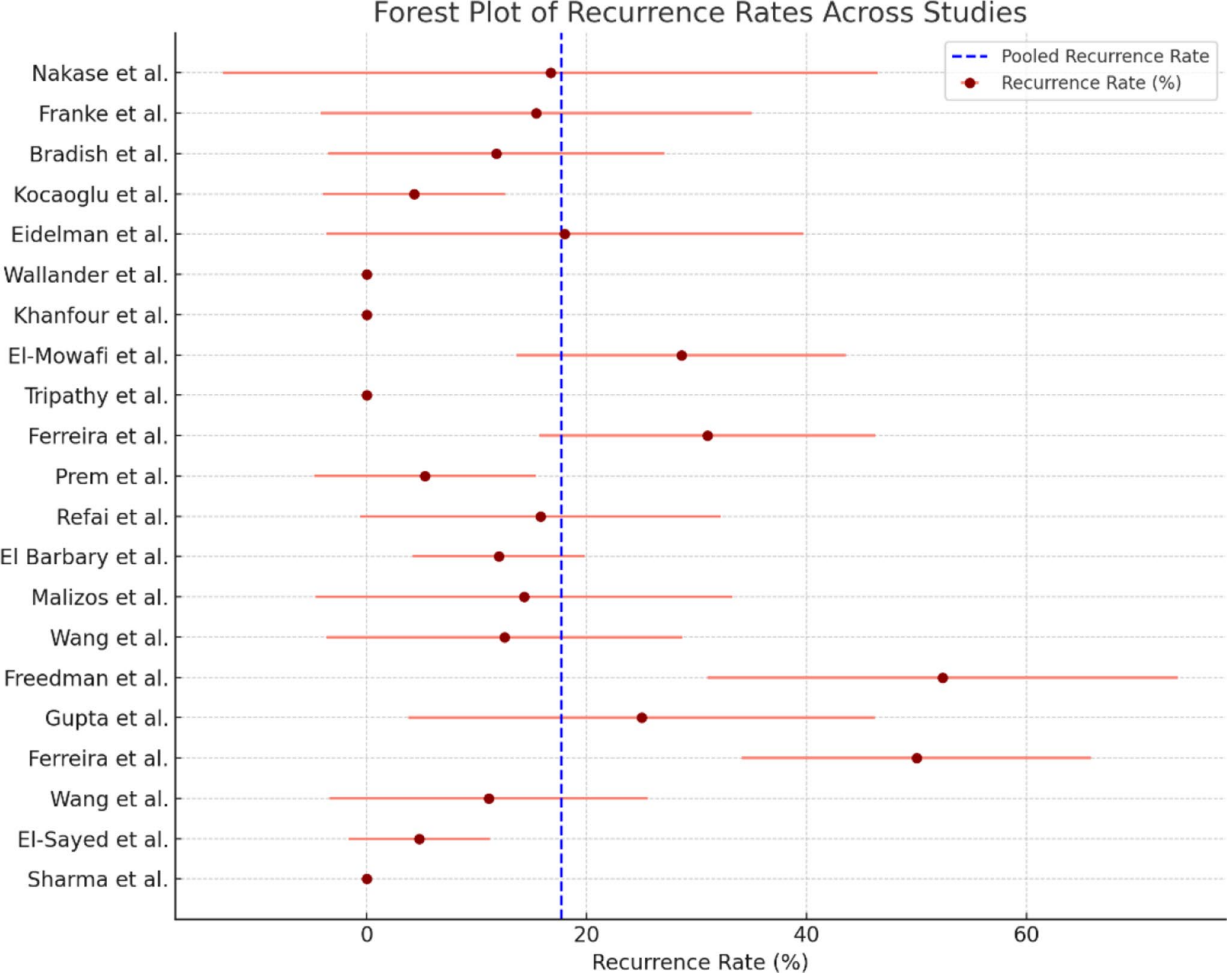
Forest Plot of Recurrence Rates Across Studies: Forest plot illustrating the recurrence rates with 95% confidence intervals for each study. The dashed line represents the pooled estimate of the recurrence rate across all studies

The forest plot (Fig. 4) illustrates the recurrence rates reported in each study and provides a clear overview of the variation in recurrence patterns. As with success rates, the low heterogeneity means that the pooled recurrence rate is a valid and consistent estimate.

### Complications

Complications were frequently reported in the included studies, highlighting the technical and clinical challenges associated with external fixation in the treatment of recurrent CF. The most commonly reported complication was pin tract infection, a known problem in patients treated with external fixation. The incidence of pin tract infection varied widely between studies, ranging from 62.5 to 93.3% of cases. For example, Sharma et al. reported the highest rate of pin tract infection, with 93.3% of patients affected. This variation may reflect differences in postoperative care, patient populations, or duration of treatment.

Other complications included toe contractures, residual deformities, and digital ischemia, which occurred with variable frequency. Cardenuto Ferreira et al. reported high rates of complications, including digital ischemia and claw toes (44.7%), highlighting the difficulties in managing severe CF deformities with external fixation. Toe contractures, although less common than pin tract infections, have been reported in several studies, with incidences ranging from 5 to 40% of patients. This type of complication is often related to inadequate soft tissue management or prolonged periods of external fixation.

**Fig. 5:**
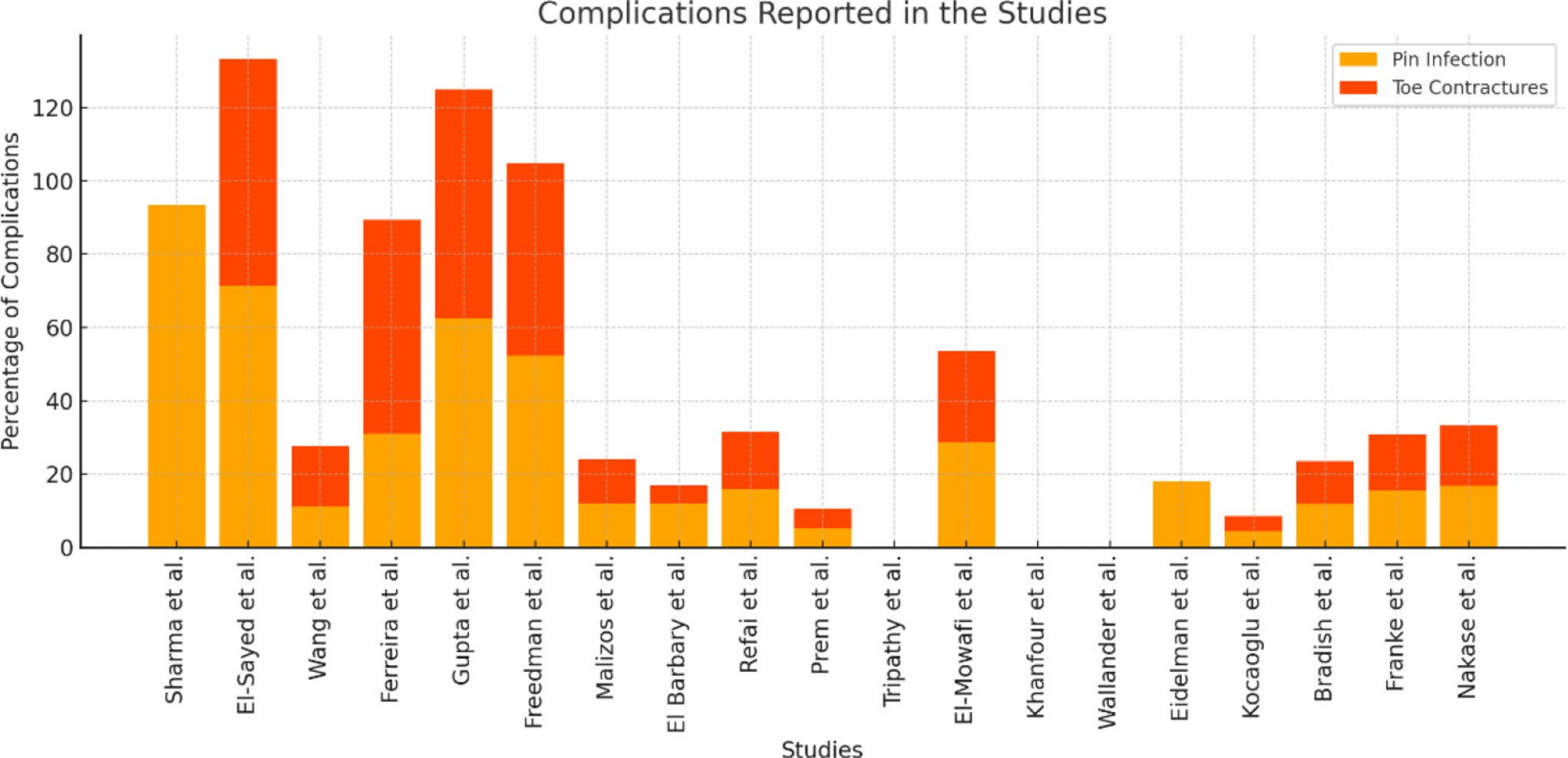
Complications Reported in the Studies: Bar chart illustrating the percentage of complications reported across the studies. Complications include pin infections and toe contractures. The studies show varying rates of complications, which may reflect differences in patient populations, surgical techniques, and follow-up duration

Figure 5 illustrates the distribution of complications across studies, showing the rates of pin tract infection and toe contracture for each study.

## Discussion

This systematic review and meta-analysis provides an in-depth examination of the efficacy and safety of external fixation, specifically the Ilizarov technique, in the correction of recurrent and complex CF deformities. The inclusion of 21 studies with 406 patients and 489 feet allowed the pooling of data to draw meaningful conclusions regarding the success rates, recurrence risks, and associated complications of this treatment modality. The overall success rate of 81.4% (95% CI: 74.5 − 88.4%) and the recurrence rate of 17.7% (95% CI: 11.3 − 24.1%) highlight the potential of external fixation as a robust method for achieving durable corrections in cases where other interventions have failed.

The high success rate observed in this review is consistent with previous findings in the literature. Burns and Sullivan [2] reported similar success rates of 76.5% in their study of adolescents with severe residual CF deformity treated with the Ilizarov technique [4]. Similarly, Kocaoglu et al. achieved plantigrade correction in 91% of patients with recurrent or neglected CF. These results confirm the effectiveness of external fixation in achieving significant corrections even in complex cases where multiple planes of deformity must be addressed simultaneously.

Ganger et al. [6] further highlighted the advantage of the Ilizarov technique in allowing for gradual correction, minimizing the risks associated with acute surgical interventions. This ability of external fixation to achieve multiplanar correction, particularly in cases of severe rigidity or previous surgical failure, underscores its utility as a versatile tool in the orthopaedic surgeon’s armamentarium. The ability to fine-tune the correction postoperatively, a feature unique to the Ilizarov technique, further enhances its effectiveness in maintaining joint congruity and achieving functional outcomes.

Despite the high success rate, the recurrence rate of 17.7% observed in this meta-analysis suggests that a subset of patients remains at risk for treatment failure and further deformity recurrence, particularly those with more complex deformities or underlying syndromic conditions. Bradish and Noor [8] reported recurrence rates of 29%, particularly in patients who had undergone multiple prior surgical procedures. This suggests that while external fixation is effective in achieving initial correction, long-term stability may require additional measures such as osteotomies or tendon transfers, particularly in patients with neuromuscular disorders or severe rigidity.

The variability in recurrence rates among the included studies may reflect differences in patient populations, treatment protocols, and follow-up periods. Studies that focused on idiopathic cases generally reported lower recurrence rates than those that included syndromic or post-traumatic deformities. Similarly, studies with shorter follow-up periods may underestimate late recurrences, which are more likely to occur years after the initial correction. The lack of standardized reporting criteria for recurrence further complicates direct comparisons between studies. In syndromic cases such as arthrogryposis, Ferreira et al. [5] found that recurrence rates could be as high as 31%, especially when external fixation was not combined with adjunctive procedures. This finding underscores the importance of tailoring the surgical approach to the patient’s underlying condition and deformity characteristics. For example, Burns and Sullivan [2] emphasized that osteotomies are often necessary to achieve permanent correction in older patients in whom bone growth has ceased.

In addition, de la Huerta [29] found that in adults with neglected CF, recurrence rates can be as high as 50%, especially in cases where the deformity is severe and has been untreated for a long time. This highlights the importance of early intervention and careful long-term follow-up to prevent late recurrences, which may not be apparent until years after initial correction. The inclusion of studies with shorter follow-up periods may have underestimated the true recurrence rate because late recurrences may not have been captured.

The importance of osteotomies and other adjunctive procedures in improving the long-term success of external fixation is well documented. Burns and Sullivan [2] noted that in older patients where bone growth has ceased, external fixation alone may not be sufficient to achieve a permanent correction. The incorporation of osteotomies, tendon lengthening, or transfers, particularly in cases of severe deformity or neuromuscular involvement, can significantly improve outcomes. Bradish and Noor [8] reported that the use of osteotomies in conjunction with external fixation reduced recurrence rates and improved functional outcomes in patients with recurrent deformity.

In addition, Atar et al. [31] highlighted the utility of tendon transfers, such as the tibialis anterior transfer, in reducing recurrence rates and improving functional outcomes. This approach is particularly beneficial in patients with muscle imbalances where external fixation alone may not be sufficient to achieve long-term stability. Combining external fixation with soft tissue procedures provides a comprehensive approach to correcting complex deformities, addressing both the bony and soft tissue components.

Pin tract infection was the most common complication reported in this meta-analysis, affecting 29.3% of patients. This finding is consistent with previous studies such as Wallander et al. [24]and Kocaoglu et al. [26] who reported infection rates of 35% and 32%, respectively. While these infections are typically superficial and manageable with antibiotics, they pose a significant risk for deeper infections that could lead to pin loosening or frame removal. Ferreira et al. [5] noted that in some cases, pin tract infections could compromise the correction and require early frame removal. The importance of rigorous postoperative care, including daily cleaning of the pin site and early detection of infection, cannot be overstated in reducing the incidence of this complication [30].

In addition to infection, toe contractures were another significant complication, particularly in patients who underwent prolonged fixation. Kocaoglu et al. [26] and Ferreira et al. [5] both reported high rates of contracture, with 44.7% of patients in the latter study. This highlights the importance of incorporating early physical therapy and soft tissue mobilization into the postoperative regimen to prevent these complications. Atar et al. [31] emphasized that the use of unconstrained frames or half-rings could help reduce the incidence of joint stiffness and contractures by allowing early mobilization.

One of the major strengths of this meta-analysis is its methodological rigor. The funnel plot analysis, which showed a symmetric distribution of studies, confirmed the absence of publication bias, a crucial aspect that increases the reliability of the results. In addition, Egger’s regression test showed no evidence of small study effects, reinforcing the validity of the pooled success and recurrence rates. These statistical controls ensure that the conclusions drawn from this meta-analysis are robust and not influenced by biases commonly found in clinical research, such as selective publication of studies with favorable results.

The low heterogeneity observed in this meta-analysis (I² < 25%) supports the conclusion that the differences in success and recurrence rates between studies are primarily due to random variation rather than substantial clinical or methodological differences. This low variability suggests that the results of this meta-analysis can be generalized to different patient populations and surgical techniques. These observations are supported by studies such as Burns and Sullivan (2004) [2] and Kocaoglu et al. [26], which also reported high success rates with relatively low variability between patients [4].

This review has limitations. The studies had different designs, populations, and follow-ups, which could have introduced bias, but overall heterogeneity was low. Most studies were retrospective, limiting control for confounding variables and increasing the risk of selection bias. While no significant publication bias was found, the possibility of unreported negative results remains. Additionally, lack of standardized outcome measures makes comparison difficult, especially for long-term complications. Furthermore, short follow-up periods may have underestimated recurrence and long-term complications. Gaber et al. [30] showed that 12 years follow up is needed to capture late recurrences and complications like arthritis. Including studies with shorter follow-up periods limits assessing the durability of external fixation. *Most studies reported aggregate data on clubfoot etiologies (idiopathic*, *syndromic*, *and post-traumatic) without distinguishing results for each subgroup This prevented us from analyzing outcomes by etiology in terms of treatment success and recurrence rates.* The lack of multicenter trials limits the generalizability of these results. Future research should focus on multicenter trials with standardized protocols and longer follow-up periods to provide more robust evidence for the use of external fixation in recurrent clubfoot.

## Conclusion

In conclusion, this systematic review and meta-analysis provides robust evidence for the efficacy of external fixation, particularly the Ilizarov technique, in the treatment of recurrent and complex CF deformities. The success and recurrence rates demonstrate that external fixation is an effective long-term correction option. However, the risk of complications highlights the need for vigilant postoperative management and early intervention. In addition, adjunctive procedures may reduce the risk of recurrence and improve functional outcomes. Future research should focus on multicenter randomized controlled trials with longer follow-up to further evaluate the long-term efficacy and safety of external fixation. The results of this meta-analysis provide a solid foundation for improving the management of recurrent CF.

## Data Availability

No datasets were generated or analysed during the current study.
